# Supplementary material for: Cooperative cobinding of synthetic and natural ligands to the nuclear receptor PPARγ
Source: eLife. 2018 Dec 21;7:e43320. doi: 10.7554/eLife.43320 (PMC6317912; doi:10.7554/eLife.43320)
Supplement: Table 4—source data 1. — Two replicate measurements (green and pink data) per ligand-bound condition (molar equivalents of 1X for rosiglitazone or edaglitazone, 2X for OA, and 3X for C9) were used for the unbiased global ITC analysis. [file elife-43320-table4-data1.pdf]

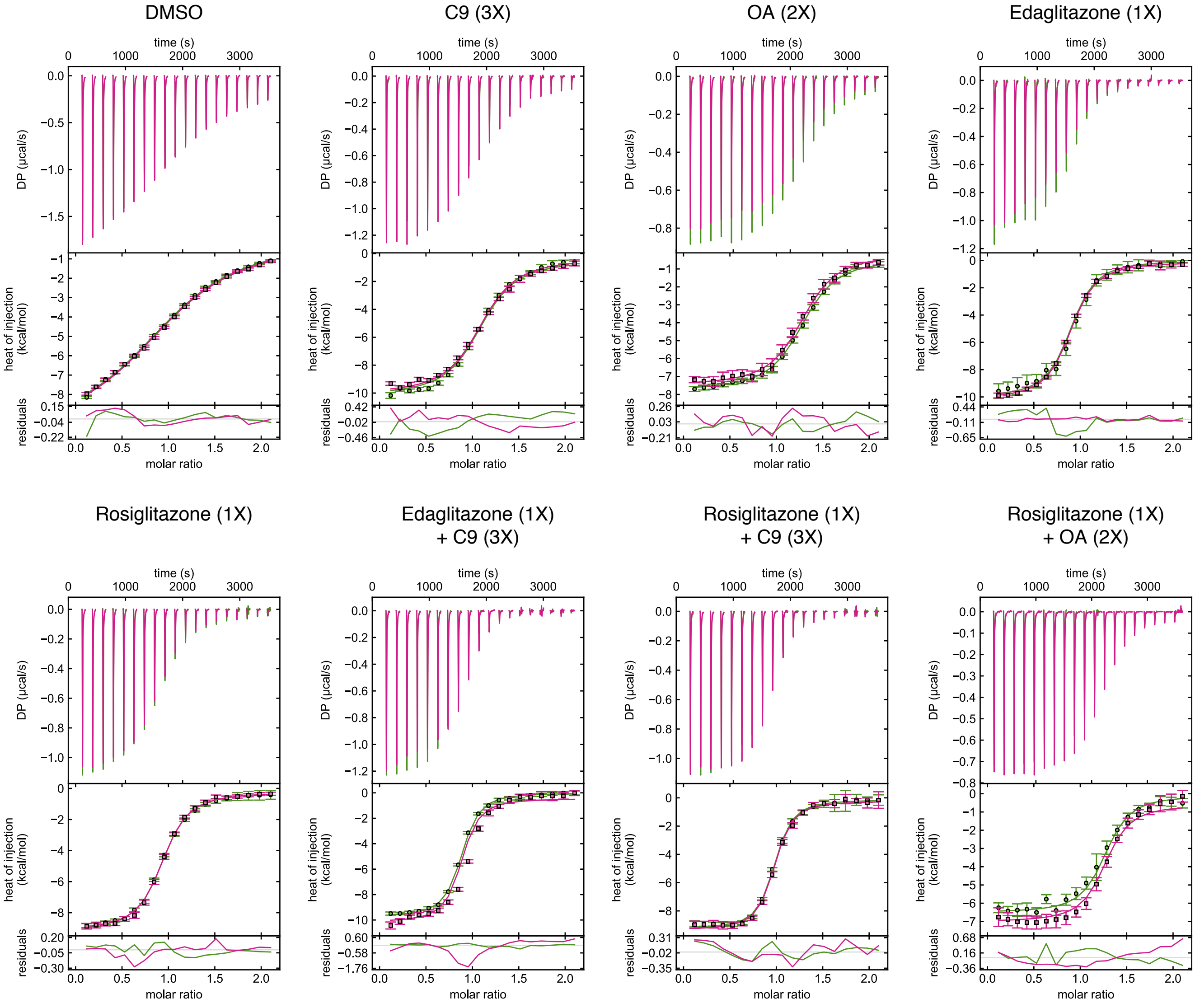
DMSO
C9 (3X)
OA (2X)
Edaglitazone (1X)
Rosiglitazone (1X)
Edaglitazone (1X) + C9 (3X)
Rosiglitazone (1X) + C9 (3X)
Rosiglitazone (1X) + OA (2X)
time (s)
DP (µcal/s)
heat of injection (kcal/mol)
residuals
molar ratio
